# Determination of Perflourooctanoic Acid Toxicity in a Human Hepatocarcinoma Cell Line

**DOI:** 10.5696/2156-9614-11.31.210909

**Published:** 2021-08-17

**Authors:** Mahmoud Abudayyak, Ezgi Öztaş, Gül Özhan

**Affiliations:** 1 Department of Pharmaceutical Toxicology, Faculty of Pharmacy, Karadeniz Technical University, Trabzon, Turkey; 2 Department of Pharmaceutical Toxicology, Faculty of Pharmacy, Istanbul University, Istanbul, Turkey

**Keywords:** perfluorooctanoic acid, oxidative stress, apoptosis, inflammation, HepG2 cells

## Abstract

**Background.:**

Perfluorooctanoic acid (PFOA) is used in different industrial and commercial products. Research shows the presence of PFOA in home dusts, tap and surface water, and in biological samples. The International Agency for Research on Cancer (IARC) has classified PFOA as a possible carcinogen for humans. The liver is thought to be a target organ of PFOA accumulation and toxicity.

**Objective.:**

Some studies have found toxic effects on the liver and related mechanisms; however, more studies are needed to better understand PFOA - induced hepatotoxicity.

**Methods.:**

In the present study, a human hepatocarcinoma cell line was exposed to PFOA for 24 hours and cell viability, apoptosis, the oxidative system and immune response were evaluated.

**Results.:**

While apoptosis was the main cell death pathway at low concentration (86.5%), the necrotic cell fraction increased with higher concentrations (46.7%). Significant changes in the reactive oxygen species (5.3-folds) glutathione (GSH) (1.7-folds) and catalase (CAT) (1.4-folds) levels were observed, as well as changes to interleukin-6 (≤1.8-fold) and interleukin-8 levels (35–40%).

**Conclusions.:**

In light of the data, PFOA is potentially hepatotoxic through the investigated pathways. The results represent a background for future in vivo mechanistic studies.

**Competing Interests.:**

The authors declare no competing financial interests.

## Introduction

The properties of perfluorooctanoic acid (PFOA), such as high stability, repellent properties and low surface tension have led to wide industrial and commercial applications.^1^ Non-stick coating for kitchen tools, protective coatings, waxes, surfactants and high-temperature lubricants are some examples of PFOA products.^1^ Perfluorooctanoic acid has been detected in home dusts, air, soil and water. Studies have found that PFOA accumulates in the human body with a high estimated half-life of approximately 3.5 years. Perfluorooctanoic acid was found in several different biological samples, including in muscles, lungs, gonads and the liver.^1^–^9^ The possible health effects have pushed many countries to restrict use of PFOA.^2^ However, humans continue to be exposed to PFOA through contaminated food and water.^1^

Perfluorooctanoic acid has been classified as a possible human carcinogen (Group 2B) by the International Agency for Research on Cancer (IARC).^1^,^6^–^7^,^10^ It is thought that chronic exposure to PFOA could be associated with cardiovascular, kidney and liver diseases, as well as pancreatic, testicular and liver cancer.^1^,^6^–^9^,^11^

Several studies have found PFOA to have hepatotoxic effects, including organ weight loss, hepatomegaly, peroxisome proliferation, epigenetic changes, and effects on lipid metabolism.^2^,^11^–^16^ The screening studies of fluorochemical production workers reported a positive relationship between PFOA exposure and serum alanine transaminase (ALT) levels, and a negative relationship with high-density lipoprotein (HDL) levels.^1^,^9^ Previous data indicate that PFOA causes the death of primary and secondary hepatocytes.^17^–^19^ Apoptosis is thought to be the main cell death pathway after exposure to PFOA.^18^–^20^ Oxidative stress in hepatocytes after exposure to PFOA has been demonstrated by both *in vitro* and *in vivo* studies.^5^,^19^ In addition, a significant increase in inflammatory response markers interleukin 6 (IL-6), cyclooxygenase-2 (Cox-2) and C-reactive protein levels was detected in hepatic tissues.^5^ However, the results are still conflicting, and the related mechanisms are controversial. Therefore, the present study aimed to elucidate some aspects of the PFOA-hepatotoxicity mechanism. The human hepatocarcinoma (HepG2) cell line, a widely used model for *in vitro* toxicity studies, was chosen for the study.^21^–^23^ 3-[4,5-Dimethylthiazol-2-yl]-2,5-diphenyl-tetrazolium bromide (MTT) and annexin V with propidium iodide (PI) apoptosis detection assays were used to evaluate cytotoxicity and the main cell death pathway, respectively. Levels of reactive oxygen species (ROS) were determined with a fluorogenic substrate using flow cytometry, and ELISA kits were used for oxidative stress endpoints, glutathione (GSH), catalase (CAT) and superoxide dismutase (SOD). Interleukin-6 & -8 (IL-6 & IL-8) detection assays for flow cytometry were used to indicate the role of inflammation response in PFOA-induced hepatotoxicity.

## Methods

Perfluorooctanoic acid (PFOA, Cat. No. 171468) at 95% was purchased from Sigma Aldrich (Munich, Germany). GSH, SOD and CAT enzyme-linked immuno sorbent assay (ELISA) assays were obtained from SunRed Biological Technology Co. (Shanghai, China). Brefeldin A, annexin V/PI apoptosis detection assay, IL-6 and IL-8 detection assay for flow cytometry were obtained from BioLegend (Koblenz, Germany). Concanavalin A (ConA, Type IV-S), hydrogen peroxide (H_2_O_2_, 30%), dimethyl sulfoxide (DMSO), sodium dodecyl sulphate (SDS), MTT and 2′,7′-dichlorodihydrofluorescein diacetate (H_2_DCF-DA) were obtained from Sigma-Aldrich (St. Louis, MO, USA). Cell culture medium and other supplements were purchased from Multicell Wisent (Quebec, Canada) and Corning (Amsterdam, The Netherlands).

**Abbreviations**
*HepG2*: Human hepatocarcinoma cell line
*IL-6*: Interleukin-6
*IL-8*: Interleukin-8
*MTT*: 3-[4,5-dimethylthiazol-2-yl]-2,5-diphenyl-tetrazolium bromide
*PFOA*: Perfluorooctanoic acid
*ROS*: Reactive oxygen species

### Cell culture and exposure conditions

Human hepatocellular carcinoma cell line (HepG2, HB-8065™) was obtained from American Type Culture Collection (ATCC, Manassas, VA, USA). The cells were cultured in Dulbecco's modified Eagle's medium (DMEM) supplemented with fetal bovine serum (10%) and antibiotic (100 U/mL penicillin and 100 mg/mL streptomycin) and incubated at 5% CO_2_ and 37°C. The cells were detached with trypsin-ethylene diamine tetraacetic acid (EDTA) either upon reaching 70–80% confluence or at the end of exposure. An appropriate number of cells were seeded into suitable-sized culture-wares according to related assay and incubated overnight for attachment. The stock solution of PFOA was prepared by dissolving in DMSO at the final concentration of 100 mM and kept at −20°C. The cell treatment was done by serial dilutions freshly made in the cell culture medium. The assays were carried out in triplicate on three independent days.

### Cytotoxicity

Cytotoxicity was determined by MTT assay. About 10^4^ cells (in 100 μL cell culture medium/well, 96-well plate) were exposed to 0–600 μmol/L PFOA for 24 h; then, 20 μL MTT (5 mg/mL in phosphate buffered saline, PBS) solution was added into each well and further incubated for 3 h. The cell culture medium was discarded, purple-colored formazan crystals were dissolved in 100 μL DMSO and the optical densities (ODs) were measured at 590 nm by Epoch microplate reader (BioTek, Germany). Inhibition of cellular viability for each concentration was calculated by comparison with control (DMSO, 1%), and the 25, 50 and 75% of inhibitor concentrations (IC_25_, IC_50_ and IC_75_) were calculated according to the equation obtained from the concentration-inhibition curve.

### Apoptotic/necrotic effects

The apoptotic and necrotic cells were stained with fluorochrome-labelled annexin V/PI; and analyzed by flow cytometer. Next, 3×10^5^ cells (in 2 mL cell culture medium, 6-well plate) exposed to 10, 25 and 50 μmol/L of PFOA for 24 h. DMSO (1%) and H_2_O_2_ (100 μmol/L) were used as negative and positive controls, respectively. Both the suspended (non-viable) and adherent (viable) cells were collected, washed twice, and re-suspended in binding buffer (100 μL). Then, fluorescein isothiocyanate (FITC) labelled-annexin V (2 μL) and PE labelled-PI (1 μL) were added and further incubated for 15 min at room temperature in the dark. Finally, the fluorescence intensities of 10^4^ cells were measured on ACEA NovoCyte flow cytometer (San Diego, USA) in FITC and PE channels at blue laser wavelength (488 nm). The results were expressed as a percentage of the total cell amount.

### Oxidative damage potential

Fluorescein isothiocyanate labelled H_2_DCF-DA was utilized to determine cellular ROS production by flow cytometer. Cells (3×10^5^ in 2 mL cell culture medium, 6-well plate) exposed to 10, 25 and 50 μmol/L PFOA for 24 h. DMSO (1%) and H_2_O_2_ (100 μmol/L) were used as negative (solvent) and positive controls, respectively. Cells were detached, re-suspended in 1 mL PBS; then, 1 μL H_2_DCF-DA (20 μmol/L in DMSO) was added, and further incubated for 30 min at room temperature in the dark. Finally, cells were washed with cold PBS, re-suspended in 150 μL BSA (in PBS, 1%); and fluorescence intensities of 10^4^ cells were measured on ACEA NovoCyte flow cytometer (San Diego, USA) in FITC channel at blue laser wavelength (488 nm). The results were expressed as percentage of median fluorescence intensity (MFI %).

### Glutathione, catalase and superoxide dismutase ELISA assays

The levels of GSH, CAT and SOD were evaluated by ELISA assays. 5×10^5^ cells (in 5 mL cell culture medium, T-25 flask) exposed to 10, 25 and 50 μmol/L of PFOA for 24 h. DMSO (1%) and H_2_O_2_ (100 μmol/L) were used as negative and positive controls, respectively. The cells were detached, counted with trypan blue; and adjusted to 10^6^ cells/mL culture medium. After the cells were centrifuged and lysed with radio immunoprecipitation assay (RIPA) cell lysis buffer, ELISA assays were performed according to the manufacturer's instructions. The Bradford method was used to measure the amount of protein in 10^6^ cells.^24^ The ODs were measured using a microplate reader (BioTek, Winooski, VT, USA). The levels of GSH, CAT and SOD were calculated using a standard calibration curve and the results were expressed as μmol/L/mg protein, μg/g protein and μg/mg protein, respectively.

### Immune response

The immune response was determined by intracellular staining of IL-6 and IL-8 by flow cytometer. The cells (10^5^ in 500 μL cell culture medium, 24-well plate) were exposed to 10, 25 and 50 μmol/L of PFOA for 24 h. DMSO (1%) and ConA (25 μg/mL) were used as negative and positive controls, respectively. In the last 4 h, Brefeldin A (0.1 %) was added into each well to block cytokine transport. The cells were detached with trypsin-EDTA, washed in PBS twice, fixed with fixation buffer for 50 min and permeabilized with permeabilization buffer for 2 min twice. Then, 1 μL FITC-labelled IL-6 (dilution: 1/100) and 1 μL PE-labelled IL-8 (dilution: 1/100) were added in permeabilization buffer and the cells were incubated for 30 min at room temperature in the dark. After centrifugation, the cells were washed with permeabilization buffer twice, and re-suspended in 150 μL ice cold PBS. Finally, fluorescence intensities of 10^4^ cells were measured on ACEA NovoCyte flow cytometer (San Diego, USA) in the FITC and Pe channels at blue laser wavelength (488 nm); and the results were expressed as MFI (%).

### Statistical analysis

Statistical analyses were carried out by one-way ANOVA followed by *post hoc* Dunnett's test using the Statistical Package for the Social Science (SPSS) software program v. 20 for Windows (SPSS Inc., Chicago, IL). The statistical evaluation was performed compared to the control group. Data was expressed as mean ± standard deviation (SD). A two-tailed *p*<0.05 was considered to indicate a statistically significant difference.

## Results

**Figure 1:**
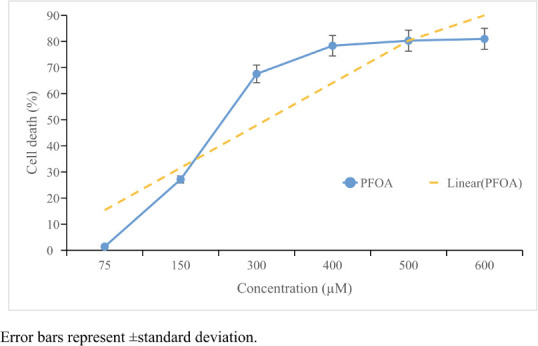
MTT results of perfluorooctanoic acid on HepG2 cells. Cell death (blue line) remained approximately 80% in higher concentrations. IC 50 value was calculated as 235.74 μM according to a logarithmic curve formula (orange line). Statistical evaluation was performed compared to the control group and results expressed as means.

Perfluorooctanoic acid decreased cell viability in a concentration-dependent manner *(Figure 1).* In the higher concentrations (≥400 μmol/L), cell death remained at approximately 80%. The IC_75_, IC_50_ and IC_25_ values were calculated to be 429.96; 235.74 and 129.3 μmol/L, respectively.

### Apoptotic/necrotic effects

**Figure 2:**
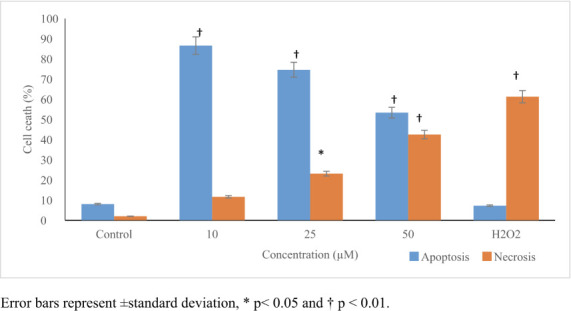
Perfluorooctanoic acid-induced cell death measured by annex in V/PI staining and analyzed by flow cytometer. Apoptotic cells were decreased in higher concentrations, while necrotic cells were increased in a concentration-dependent manner. 100 μM H_2_O_2_ , as positive control, induced necrosis rather than apoptosis. The results were presented as a percentage of the total cell amount. Control cells were exposed to 1% DMSO. Statistical evaluation was performed compared to the control group and results are expressed as means.

The annexin V/PI assay results show that PFOA induced cell death by both the apoptosis and necrosis pathways. However, the apoptotic cell percentages (86.5%, 27-fold) decreased (53.3%, 15-fold) with the increase in PFOA concentration, while the necrotic cells ratios (42.5%, ≤50-fold at 50 μmol/L) increased in a concentration-dependent manner *(Figure 2).*

### Oxidative damage potential

**Figure 3:**
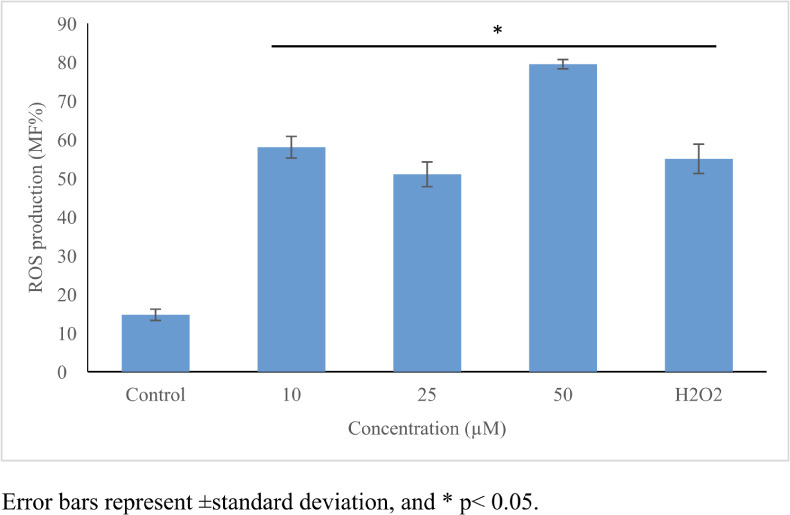
Oxidative stress inducing potential of perfluorooctanoic acid on HepG2 cells. Perfluorooctanoic acid-induced ROS production was tested in all concentrations. 100 μM H_2_O_2_ was used as positive control. Control cells were exposed to 1% DMSO. Statistical evaluation was performed compared to the control group and results expressed as means.

**Figure 4:**
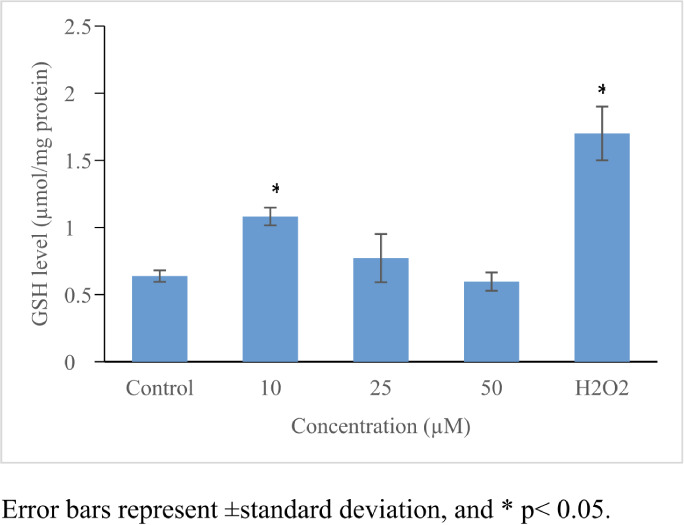
Oxidative stress inducing potential of PFOA on HepG2 cells. Perfluorooctanoic acid increased GSH level only in the 10 μM concentration. 100 μM H_2_O_2_ was used as positive control. Control cells were exposed to 1% DMSO. Statistical evaluation was performed compared to the control group and results expressed as means.

**Figure 5:**
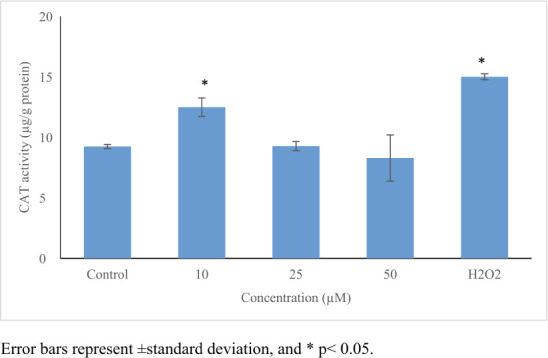
Oxidative stress inducing potential of perfluorooctanoic acid on HepG2 cells. Perfluorooctanoic acid increased CAT activity only in 10 μM; 100 μM H_2_O_2_ was used as positive control. Control cells were exposed to 1% DMSO. Statistical evaluation was performed compared to the control group and results expressed as means.

**Figure 6—:**
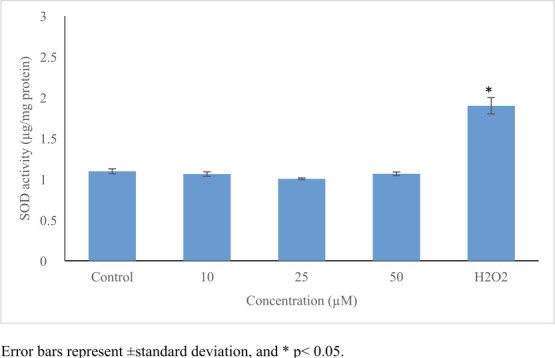
Oxidative stress-inducing potential of perfluorooctanoic acid on HepG2 cells Superoxide dismutase activity remained unchanged after exposure to PFOA. 100 μM H_2_O_2_ was used as positive control. Control cells were exposed to 1% DMSO. Statistical evaluation was performed compared to the control group and results expressed as means.

Perfluorooctanoic acid significantly increased ROS production (≤5.3-fold, *p*≤0.05) *(Table 1) (Figure 3).* An increase in GSH level (1.7-fold) *(Figure 4)* and in CAT activity (1.4-fold) *(Figure 5)* was noticed only at 10 μmol/L, while no significant changes were noticed in SOD activity *(Table 1, Figures 3 and 6).*

**Table 1—:** Oxidative Stress-Inducing Potential of Perfluorooctanoic Acid on HepG2 Cells. H_2_O_2_ was used as a positive control. Control cells were exposed to 1% DMSO. The statistical evaluation was performed compared to control group. The results are expressed as the means.

**Concentration**	**ROS (MIF %)**	**p values**	**SD**	**CI**
Control	14.7	-	1.46	12.01 – 27.69
10	58.0*	0.04	2.80	31.14 – 42.27
25	51.0*	0.04	3.19	23.43 – 70.20
50	79.4*	0.03	1.21	71.19 – 84.19
H_2_O_2_	55.0*	0.04	3.78	21.77 – 38.95
	**CAT (μg/g protein)**			
Control	9.2	-	0.17	8.98 – 9.42
10	12.5*	0.04	0.76	11.72 – 13.26
25	9.3	0.07	0.37	8.43 – 9.27
50	8.3	0.09	1.91	5.77 – 10.39
H_2_O_2_	15.0*	0.04	0.25	11.03 – 11.58
	**GSH (μmol/mg protein)**			
Control	0.6	-	0.04	0.59 – 0.68
10	1.1*	0.03	0.07	1.03 – 1.08
25	0.8	0.07	0.18	0.60 – 1.01
50	0.6	0.08	0.07	0.53 – 0.68
H_2_O_2_	1.7*	0.04	0.20	0.66 – 1.70
	**SOD (μg/g protein)**			
Control	1.1	-	0.03	1.05 – 1.19
10	1.1	0.11	0.03	1.03 – 1.09
25	1.0	0.07	0.01	0.99 – 1.02
50	1.1	0.09	0.02	1.04 – 1.09
H_2_O_2_	1.9*	0.04	0.10	0.88 – 1.90

Abbreviations: CI, confidence interval; SD, standard deviation.

^*^p<0.05.

### Immune response

**Figure 7:**
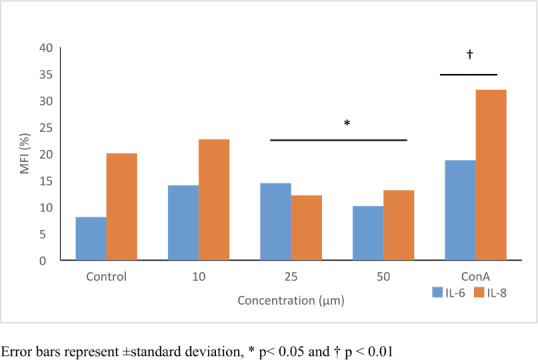
IL-6 and IL-8 producing cells analyzed by flow cytometer. Perfluorooctanoic acid induced IL-6 in all concentrations tested; however, IL-8 levels were decreased in 25 and 50 μM. 25 μg/mL ConA was used as positive control and found to induce both IL-6 and IL-8 levels. Control cells were exposed to 1% DMSO. Statistical evaluation was performed compared to the control group and results expressed as means.

The flowcytometric evaluation of IL-6 & -8 show that PFOA significantly induced IL-6 levels (≤1.8-fold; *p*≤0.05) but suppressed IL-8 levels in 25 μmol/L (40%) and 50 μmol/L (35 %). (*p*≤0.05) *(Table 2, Figure 7).*

**Table 2:** Perfluorooctanoic acid effect on IL-6 and IL-8 Levels in HepG2 Cells after 24-h Exposure. Concanavalin A was used as positive control. Control cells were exposed to 1% DMSO. Statistical evaluation was performed compared to the control group. Results are expressed as means.

**Concentration**	**IL-6 (MIF %)**	**p values**	**SD**	**CI**
Control	8.09	-	0.08	7.68 – 8.09
10	14.05	0.09	1.60	4.63 – 23.14
25	14.47*	0.04	1.03	11.85 – 17.17
50	10.17*	0.03	0.90	8.91 – 11.43
ConA	18.75**	0.003	2.10	11.04 – 24.54
	**IL-8 (MIF %)**			
Control	20.08	-	1.53	19.78 – 20.37
10	22.69	0.12	2.91	20.17 – 25.20
25	12.19*	0.03	1.42	9.10 – 17.85
50	13.14*	0.04	1.75	10.71 – 14.75
ConA	32.00**	0.002	0.90	29.08 – 34.27

Abbreviations: CI, confidence interval; ConA, concanavalin A; SD, standard deviation.

^*^p<0.05 and

^**^p<0.01.

## Discussion

The potential toxicity of PFOA on different systems has been previously reported and has led many countries to restrict its use. Nevertheless, humans are still exposed to PFOA through water, air and food,^1^–^3^,^25^ even though a possible relationship between PFOA exposure and cardiovascular, pancreatic, testicular and liver disease has been reported.^1^,^6^–^9^ Previous studies showed that PFOA accumulates in the liver of experimental animals, causing an increase in liver weight and enlargement of hepatocytes with necrotic features; cytoplasmic vacuolation and hepatocytic hypertrophy; acidophilic cytoplasm; and multifocal coagulation and liquefaction necrosis.^5^,^26^–^30^ Alteration of the metabolic profile by PFOA, especially glucose and fatty acid metabolisms, and indirectly, mitochondrial dysfunction, are also thought to induce hepatotoxicity and inflammation response in the liver.^18^,^31^–^34^

Increases were reported in oxidative stress markers and in some inflammatory markers (IL-6, Cox-2, C-relative protein *etc.*) in the liver tissue of PFOA-exposed animals.^5^,^35^ Perfluorooctanoic acid induced oxidative stress, cytotoxicity, apoptosis, cell cycle arrest in human hepatic primary (L-02),^36^ *Oreochromis niloticus* primary liver,^37^ Vero,^27^ human hepatocyte HL-7702,^18^ and fish liver cells.^20^ Thorup *et al.* 2010 evaluated the genotoxicity of PFOA and other perfluorinated chemicals in the HepG2 cell line and found that PFOA increased ROS levels in a dose-independent manner.^38^ On the other hand, Yang (2010) reported that PFOA at concentrations up to 100 mg/L did not induce a significant change in CAT, SOD or glutathione peroxidase (GPx) activities.^39^ The results of the present study show that PFOA caused cell death and the half-maximal inhibitory concentration (IC_50_) value was calculated to be 235.74 μmol/L. In contrast to previous studies, a decrease in the apoptotic cell percentages (86.5%, 27-fold) and increase in necrotic cells ratios (42.5%, ≤50-fold at 50 μmol/L) were reported in a concentration-dependent manner *(Figure 2).* It can be suggested that the main mechanism of cell death induced by PFOA is apoptosis in lower concentrations, whereas necrosis plays a significant role as a cell death pathway in higher concentrations.

Guruge *et al.* 2006 observed the gene expression profile in the liver tissue of PFOA-exposed rats at 1–15 mg/kg; they reported alterations in expression of more than 800 genes at 10 mg/kg dose, with up regulation of 106 genes and down regulation of 38 genes in all tested groups.^40^ Some of the altered genes are responsible for lipid metabolism, cell communication, apoptosis and hormone regulatory pathways. In addition, PFOA was reported to increase mRNA expression levels of IL-6, IL-1β and tumor necrosis factor alpha (TNF-α) pro-inflammatory cytokines in *Oryzias latipes* liver cells.^40^ Similarly, Yang (2010) reported upregulation of PPAR-α expression and an increase in peroxisomal acyl-CoA oxidase activity.^39^ In the present study, PFOA increased IL-6 levels but at the same time decreased IL-8 levels *(Figure 7).* This result may be connected to the TGFβ1-induced Smad2 phosphorylation pathway, which results in an increase of IL-6 and suppression of IL-8.^41^

## Conclusions

The results of the present study indicate that PFOA induces cell death by both necrosis and apoptosis pathways; however, necrosis seems to play a larger role at higher concentrations. Increases in ROS levels, along with changes in CAT and GSH, highlight the role of oxidative stress, while an increase in IL-6 and decrease in IL-8 levels indicate an immune response role. The results, while confirming some of the previous data, disaffirm some others, which shows the need for further mechanistic studies to elucidate the differences and mechanism of PFOA.
